# Assigning crystallographic electron densities with free energy calculations—The case of the fluoride channel Fluc

**DOI:** 10.1371/journal.pone.0196751

**Published:** 2018-05-17

**Authors:** Igor Ariz-Extreme, Jochen S. Hub

**Affiliations:** Institute for Microbiology and Genetics, University of Goettingen, Göttingen, Germany; Wake Forest University, UNITED STATES

## Abstract

Approximately 90% of the structures in the Protein Data Bank (PDB) were obtained by X-ray crystallography or electron microscopy. Whereas the overall quality of structure is considered high, thanks to a wide range of tools for structure validation, uncertainties may arise from density maps of small molecules, such as organic ligands, ions or water, which are non-covalently bound to the biomolecules. Even with some experience and chemical intuition, the assignment of such disconnected electron densities is often far from obvious. In this study, we suggest the use of molecular dynamics (MD) simulations and free energy calculations, which are well-established computational methods, to aid in the assignment of ambiguous disconnected electron densities. Specifically, estimates of (i) relative binding affinities, for instance between an ion and water, (ii) absolute binding free energies, i.e., free energies for transferring a solute from bulk solvent to a binding site, and (iii) stability assessments during equilibrium simulations may reveal the most plausible assignments. We illustrate this strategy using the crystal structure of the fluoride specific channel (Fluc), which contains five disconnected electron densities previously interpreted as four fluoride and one sodium ion. The simulations support the assignment of the sodium ion. In contrast, calculations of relative and absolute binding free energies as well as stability assessments during free MD simulations suggest that four of the densities represent water molecules instead of fluoride. The assignment of water is compatible with the loss of these densities in the non-conductive F82I/F85I mutant of Fluc. We critically discuss the role of the ion force fields for the calculations presented here. Overall, these findings indicate that MD simulations and free energy calculations are helpful tools for modeling water and ions into crystallographic density maps.

## Introduction

Roughly 130000 macromolecular structures have been deposited in the Protein Data Bank (PDB) by 2017 [1, 2], of which more than 120000 have been determined using X-ray crystallography or cryo-electron microscopy. Overall, the quality of macromolecular structures in the PDB is very high, in part thanks to protein modeling and validation routines such as COOT, MolProbity, Procheck, NQ-Flipper, WHAT_CHECK, and several others [3–7]. Besides, the project PDB_REDO provides a protein re-refinement procedure in which the entire content of the PDB is re-evaluated [8]. However, whereas crystal structure analysis has been partially automated, the interpretation of certain electron density maps still needs a significant amount of human supervision and expertise [9], specially for non-covalently bound molecules such as organic ligands, water, and ions, which are usually more disordered than nearby residues [10, 11]. For certain ions, the anomalous scattering signal may guide the correct assignment. However, because the anomalous signal is often weak, this approach is not always applicable.

For instance, since the structure of K^+^ channel KcsA from *Streptomyces lividans* was obtained in 1998 (PDB ID 1BL8) [12], the interpretation of the electron densities found in the selectivity filter has triggered lively debates. Whether the electron densities represent two cations intercalated with water molecules [13, 14] or four cations in a row [15] has remained controversial. As another example of ambiguous electron densities may serve the obsolete models of *β*-glucosidase that contained 252 and 199 sodium ions, respectively, later mostly reassigned to water molecules (PDB IDs 3FJ0 and 3FIY, superseded by 4HZ8 and 4HZ6, respectively) [16]. Further, in the model of cyclic bovine pancreatic trypsin inhibitor (PDB ID 1K6U), a single water molecule should have been modeled instead of ethylene glycol molecule [17, 18]. Some tools such as PURY [19], CheckMyMetal [20], *Twilight* [21], or PHENIX [22] have been developed in an attempt to resolve the nature of isolated electron densities.

Molecular dynamics (MD) simulations are an established tool for refining atomic structures against experimental data, as for instance implemented in the CNS and MDFF/xMDFF software suites [23–26]. Further, Malde and Mark (2011) [27] showed that MD simulations and free energy calculations provide a useful framework to assign ligands in X-ray crystal complexes. For example, this proved to be a succesful strategy for determining the stereochemistry of the HIV-1 protease inhibitor JG-365 (PDB ID 7HVP) [28, 29] or the orientation of the ligand L-Serine in the binding pocket of *Pyrococcus abyssi* threonil-tRNA synthetase (PDB ID 2HKZ) [30]. Given the constant improvement of hardware and algorithms, MD simulations and free energy calculations are increasingly resorted techniques that may help to solve ambiguous assignments of electron densities. Because ions and water play key functional roles in many proteins, the correct assignment of such densities may aid the understanding of protein function at molecular level. With this aim in mind, we here investigate electron densities of the fluoride extruding Fluc channel.

Fluc is a family of small membrane proteins found in a wide range of unicellular organisms [31–33]. The expression of Fluc channels is controlled by a fluoride-responsive riboswitch, and their function is to allow the export of fluoride anions, thereby keeping the cytoplasmic F^−^ concentration below the enzyme inhibitory levels [34, 35]. Electrophysiological evidence states that Fluc channels are highly selective for fluoride anions over chloride or sodium ions, with a time- and voltage-independent current, reaching ∼ 10^7^ fluoride ions/sec at −200 mV [32]. However, the molecular details about the permeation mechanism remain unknown.

**Fig 1:**
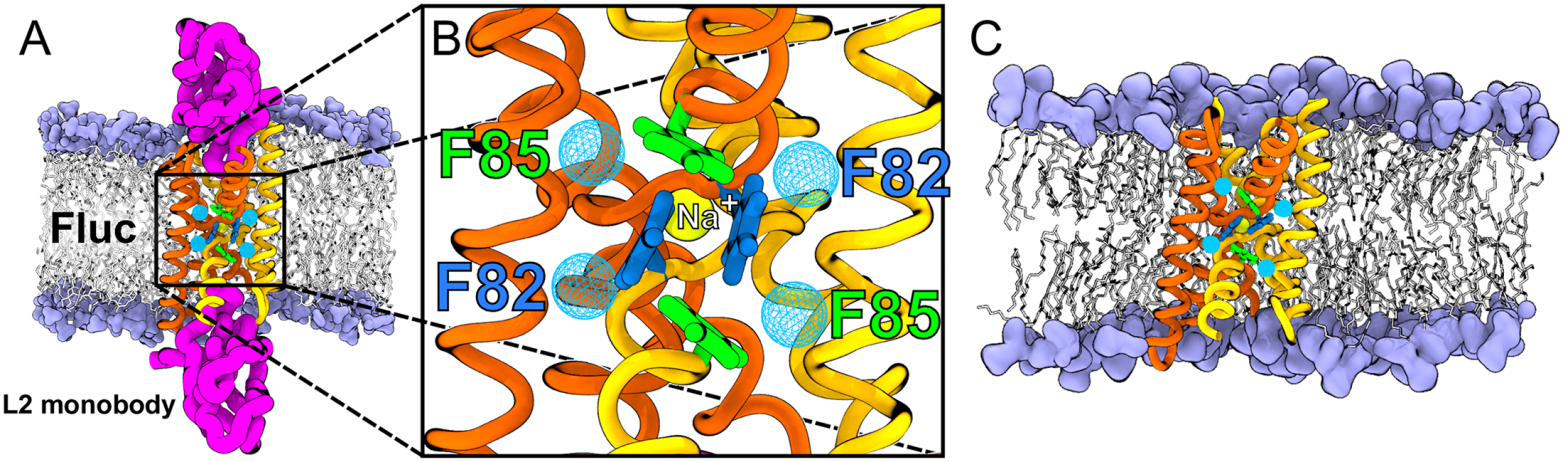
Fluc channel simulation system and structure. (A) Fluc channel simulation system with blocking L2 monobodies. Fluc is depicted as orange and yellow ribbons, L2 monobodies are showed as pink thick ribbons, lipids heads are shown as purple volume and lipid tails as white sticks. Water molecules not shown for the sake of clarity. (B) Close-up of Fluc channel electron densities (cyan) at F82 (blue) and F85 (green) sites. The sodium ion at the TM3 site is also highlighted in yellow. (C) Fluc channel simulation system without blocking L2 monobodies.

Fluc channels arrange in an antiparallel homodimeric double-barreled structure that is new among membrane proteins (PDB ID 5NKQ) [36]. The crystal structure of the *Bordetella pertussis* Fluc (Fluc-*Bpe*) is blocked by L2 monobodies (Fig 1A) [37, 38], and reveals five intriguing disconnected electron densities inside the protein (Fig 1B). The first of the electron densities is coordinated by four carbonyl groups of residues G77 and T80 at the break of the two transmembrane helices 3 (TM3 site). This density was assigned to a structural sodium ion although this type of tetrahedral coordination is only seen in ∼ 3.5% of sodium binding sites, according to the MetalPDB database [39]. The remaining four electron densities are surrounded by the electropositive side chains of N43, S108, S112, F82, and F85. Because this environment is compatible with a halide coordination shell, these four electron densities were “provisionally” identified as fluoride anions, as emphasized the authors of the 5NKQ structure [36]. Interestingly, Last *et al.* showed that crystal structures of the non-conductive F80I and F83I mutants of the *Escherichia coli* Fluc (PDB ID 5KBN and 5KOM, respectively), lack the electron densities associated to F80 and F83 residues (analogues of F82 and F85 residues in Fluc-*Bpe*, respectively) [40]. In other words, the loss of electron densities at the F80 and F83 sites correlates with a loss of protein function. This intriguing finding was taken as an indication that the electron densities at the F82 and F85 sites represent functionally relevant fluoride binding sites.

In this study, taking Fluc as an example, we illustrate how free MD simulations and free-energy calculations may help with the assignment of ambiguous disconnected electron densities. For the TM3 site, we find that variations in the structural stability during free simulation indeed favor sodium over water, in line with the original assignments. For the four densities at the F82 and F85 residues, however, free-energy calculations as well as stability assessments strongly favor water over fluoride.

## Materials and methods

### System setup for MD simulations

#### Amber ff99SB*-ILDN forcefield

Initial coordinates of Fluc-*Bpe* were taken from the Protein Data Bank (PDB ID 5FXB, now superseded by 5NKQ) [36]. Initial coordinates for the lipid membrane were taken from a 1 ns equilibrium simulation of a hydrated membrane of 328 1-palmitoyl-2-oleoyl-*sn*-glycero-3-phosphocholine (POPC) lipids. The protein was embedded in the membrane with the *g_membed* software [41]. The simulation box was filled by explicit TIP3P [42] water and neutralized with counter ions. Four different simulation systems were set up either of the wild-type (WT) or of the F82I/F85I mutant, each including or excluding the blocking L2 monobodies (which were present in the crystal). The final simulation system with blocking L2 monobodies contained one homodimer Fluc channel, two blocking L2 monobodies, 188 POPC molecules, 17685 water molecules, 6 sodium ions (including the structural Na^+^), and 4 fluoride ions (Fig 1A). Similarly, the final simulation system without L2 monobodies contained one homodimer Fluc channel 204 POPC molecules, 18052 water molecules, 1 structural sodium ion, and 7 fluoride ions (Fig 1C). The ion parameters were taken from Joung and Cheatham (2008) [43]. The F82I/F85I mutant was generated using PyMOL [44].

Interactions of the protein atoms were described by the Amber ff99SB*-ILDN forcefield [45], and lipid parameters were taken from Berger *et al.* and Cordomi *et al.* [46, 47]. The simulations were carried out with the GROMACS simulation software (version 5.1.4) [48]. Electrostatic interactions were calculated at every step with the particle-mesh Ewald method [49]. Short-range repulsive and attractive dispersion interactions were described by a Lennard-Jones (LJ) potential, with a cut-off at 1 nm. The geometry of water molecules was constrained with the SETTLE algorithm [50], and all other bond lengths were constrained with LINCS [51]. Hydrogen atoms of the protein were constructed as virtual sites, allowing a 4 fs time step [52].

The simulation temperature was controlled at 300 K using velocity rescaling (*τ* = 2.5 ps) [53], and the pressure was kept at 1 bar with a semiisotropic Berendsen barostat (*τ* = 2 ps) [54]. The systems were equilibrated for 20 ns with position restraints on the protein heavy atoms and on the molecules placed at the electron density sites (fluoride or water molecules at F82 site and F85 site, see Fig 1B), with a force constant of 1000 kJ mol^−1^nm^−2^. After that, the systems were equilibrated for 400 ns with position restraints (*k* = 1000 kJ mol^−1^nm^−2^) on the protein C_*α*_ atoms and on the molecules placed at F82 and F85 sites before production.

To investigate the stability of the fluoride and water ions assigned to the densities at the F82 and F85, multiple free production simulations were started from random time points of the 400 ns equilibration simulations. Here, four different system types were defined for free simulations: (i) four fluoride ions at F82 and F85 sites / without monobodies; (ii) fluoride ions at F82 and F85 sites / with monobodies; (iii) four water at F82 and F85 sites / without monobodies; (iv) water at F82 and F85 sites / with monobodies. For systems with or without blocking L2 monobodies, we carried out 10 replica simulations of 80 ns each or 20 replica simulations of 40 ns each, respectively. All the production simulations were carried out without position restraints, and using the parameters described above.

#### CHARMM36 forcefield

To exclude that the qualitative results depend on the applied protein force field, another set of simulation systems were setup using the CHARMM force field family [55, 56]. Setup of the system was identical to the setup for the Amber force field (see above), except that Fluc was solvated in a membrane of 1,2-dipalmitoyl-*sn*-phosphatidilcholine (DPPC) instead of POPC. The final CHARMM simulation system contained one homodimer of WT Fluc, 228 DPPC molecules, 13273 TIP3P water molecules, 1 structural sodium ion, 3 chloride ions, and 4 fluoride ions. The fluoride ion parameters were again taken from Joung and Cheatham (2008) [43], and the sodium and chloride parameters correspond to the CHARMM36 defaults.

Interactions of the protein atoms and lipid atoms were described by the CHARMM36 forcefield [55, 56]. The simulations were carried out with the GROMACS simulation software (version 5.1.4) [48]. Electrostatic interactions were calculated at every step with the particle-mesh Ewald method [49], and using a cut-off at 1.2 nm for LJ interactions. The geometry of water molecules was constrained with the SETTLE algorithm [50], and all bonds of hydrogen atoms were constrained with LINCS [51]. A 2 fs timestep was used. The simulation temperature was controlled at 323 K using velocity rescaling (*τ* = 2.5 ps) [53], and the pressure was kept at 1 bar with a semiisotropic Berendsen barostat (*τ* = 2 ps) [54]. The system was equilibrated for 20 ns with position restraints on the protein heavy atoms and on the fluoride ions placed at the F82 and F85 sites with a force constant of 1000 kJ mol^−1^nm^−2^. Subsequently, the system was equilibrated for 5 ns with position restraints on the protein C_*α*_ atoms and on the fluoride ions placed at F82 and F85 sites before production.

Finally, by assigning new random velocities, we generated 10 independent replicas from the last snapshot taken from the 5 ns equilibration trajectory, and we ran each replica for 40 ns.

#### Polarizable DRUDE forcefield

To furter exclude that polarizability could affect the qualitative results, a simulation system was set up using the polarizable DRUDE forcefield [57–59]. Here, initial coordinates for the lipid membrane were taken from a 3 ns equilibrium simulation of a hydrated membrane of 256 DPPC lipids. The protein was again embedded in the membrane with the *g_membed* software [41]. The simulation box was filled by explicit SWM4-NDP [60] water and neutralized with counter ions. The final simulation system contained one homodimer WT Fluc channel, 228 DPPC molecules, 10181 water molecules, 1 sodium ion, and 7 fluoride ions. The ion parameters were taken from Yu *et al.* (2010) [61].

Interactions of the protein atoms were described by the polarizable DRUDE forcefield [57–59], and lipid parameters were taken from Chowdhary *et al.* [62]. The GROMACS DPPC topology file was generated from original DRUDE-CHARMM topology files using TopoGromacs [63]. The simulations were carried out with the GROMACS simulation software (version DRUDE) [64, 65]. Electrostatic interactions were calculated at every step with the particle-mesh Ewald method [49], and using a cut-off at 1.2 nm for LJ interactions. The geometry of water molecules was constrained with the SETTLE algorithm [50], and all other bond lengths were left unconstrained.

To avoid polarization catastrophes, the system was carefully equilibrated with multi-step protocol. First, we equilibrated the simulation system using the self-consistent field (SCF) approach, as implemented by Van Maaren and Van der Spoel [64]. The simulation temperature was controlled at 323 K using velocity rescaling (*τ* = 0.1 ps) [53], and the pressure was kept at 1 bar with a semiisotropic Berendsen barostat (*τ* = 1 ps) [54]. Using a 0.2 fs time step, the system was equilibrated for 37 ps with position restraints on the protein heavy atoms (force constant of 1000 kJ mol^−1^nm^−2^). Next, the system was equilibrated for 800 ps with position restraints on the protein C_*α*_ atoms and on the fluoride ions placed at the F82 and F85 sites. In this last equilibration, the simulation temperature was controlled at 323 K for the non-drude atoms, and at 1 K for the drude particles, using the Nose-Hoover thermostat (*τ* = 0.1 ps, and *τ* = 0.005 ps, respectively) [66, 67]. No pressure-coupling was used, and a 1 fs time step was used with the extended Lagrangian dynamics implemented in Gromacs by Lemkul *et al.* [65].

Finally, we generated 10 different replicas from the last snapshot taken from the 800 ps equilibration trajectory and ran each of them for 10 ns.

### Free-energy calculations of absolute and relative binding

**Fig 2:**
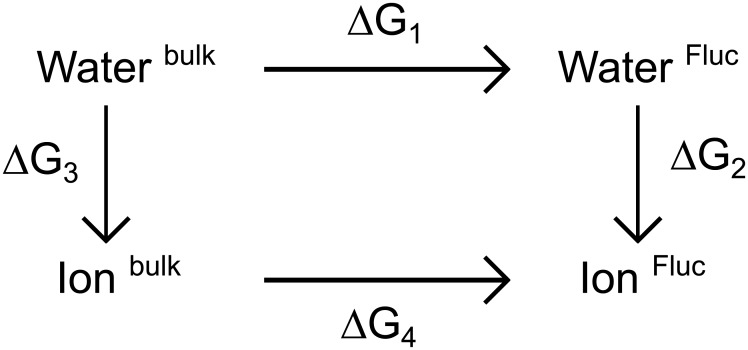
Alchemical transformation thermodynamic cycle. From the thermodynamic cycle, the binding free energy difference ΔΔ*G*_bind_ = Δ*G*_1_ − Δ*G*_4_ between the ion-bound Fluc channel and the water-bound Fluc channel can be calculated via Δ*G*_3_ − Δ*G*_2_.

We carried out two sets of free energy calculations: First, to compute *relative* binding free energies, an ion was alchemically transformed into a water molecule at the binding site while simultaneously transforming a water molecule into an ion in bulk solvent. According to a thermodynamic cycle (Fig 2), the associated free energy difference equals the free energy difference ΔΔ*G*_bind_ of binding an ion versus a water molecule into the binding site. A negative ΔΔ*G*_bind_ implies that a water molecule is more likely to translocate from solvent to the binding site as compared to the ion. More quantitatively, in equilibrium, the probabilities of finding a water molecule $P_{\text{bind}}^{\text{wat}}$ or of finding an ion $P_{\text{bind}}^{\text{ion}}$ at the binding site are related via $P_{\text{bind}}^{\text{wat}}/P_{\text{bind}}^{\text{ion}}=c_{\text{wat}}/c_{\text{ion}}\times \text{exp}\left(-\Delta \Delta G_{\text{bind}}/k_{\text{B}}T\right)$, where *c*_wat_ and *c*_ion_ denote the water and ion concentrations in the solvent, *k*_B_ is the Boltzmann constant, and *T* the absolute temperature. Hence, the ΔΔ*G*_bind_ is in principle the key quantity to detect the correct assignment of the ambiguous electron densities. However, ΔΔ*G*_bind_ is associated with an increased uncertainty, mainly because force fields might not represent ion-protein interactions as accurately as water-protein interactions [68–72].

Second, absolute binding free energies Δ*G*_bind_ for water molecules were computed, that is, free energy for translocating a water molecule from solvent into the binding site. Because the probability of finding a water molecule at the binding site is proportional to exp(−Δ*G*_bind_/*k*_B_*T*), the assignment of a water molecule to an ambiguous electron density is only plausible if Δ*G*_bind_ is negative or at least close to zero.

All free energy calculations were conducted using discrete thermodynamic integration (TI) along an alchemical reaction coordinate λ, where λ = 0 and λ = 1 denote the initial and the final state. During TI calculations, a time step of 2 fs was applied, and the temperature was controlled at 300 K using a stochastic dynamics integration scheme (*τ* = 0.1 ps) [73]. Starting configurations were taken from the final snapshot of the 20 ns equilibrium simulation. All the other simulation parameters were identical to the equilibrium simulations.

#### Relative binding free-energy computational details

The parameter λ was chosen such that λ = 0 described the state with the ion in the protein (fluoride at F82 or at F85 site, or sodium TM3 site) while having a water in bulk solvent. λ = 1 described the ion (fluoride or sodium) in bulk solvent while having a water molecule at the F82, F85, or TM3 site. The free energy calculations with fluoride were done in the presence or absence of blocking L2 monobodies, while the free energy calculations with sodium were done only in presence of monobodies. To keep the structure close to the crystal structure, position restraining potentials (*k* = 1000 kJ mol^−1^nm^−2^) were applied on the protein C_*α*_ atoms and on the ion and water molecules that were alchemically transformed. Here, the molecule in solvent was restrained at a position as far as possible from the protein and membrane. To avoid numerical instabilities close to λ = 0 and λ = 1, soft-core potentials were used for both electrostatics and Lennard-Jones interactions with *α* = 0.5, *σ* = 0.3 and a soft-core power of 1. The transformation from λ = 0 to λ = 1 was done in 41 λ-steps, where each λ-steps was simulated for 10 ns. Derivatives of the Hamiltonian with respect to λ were recorded at every step. Free energy differences were calculated from

$$\begin{array}{c} \Delta \Delta G_{\text{bind}}=\int_{0}^{1}\langle \frac{\delta H}{\delta \lambda }\rangle \text{d}\lambda \end{array}$$ (1)

where 〈⋅〉 denote the average over the trajectory, after removing the first 50 ps for equilibration.

#### Absolute binding free energy computational details

**Fig 3:**
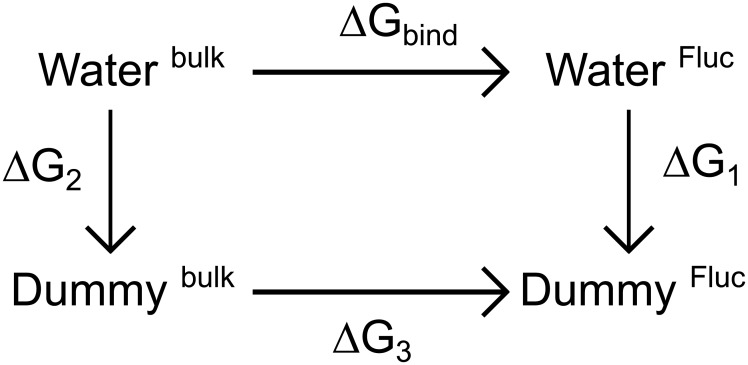
Absolute binding free energy thermodynamic cycle. From the thermodynamic cycle, the absolute binding free energy is calculated via Δ*G*_bind_ = Δ*G*_2_ − Δ*G*_1_, where Δ*G*_3_ ≡ 0.

Absolute binding free energies Δ*G*_bind_ of water were calculated using TI following the scheme shown in Fig 3. Accordingly, Δ*G*_bind_ was calculated from the difference in free energy between (i) turning off the interactions of a water molecule in a pure water box (Δ*G*_2_ in Fig 3), and (ii) turning off the interactions of a water molecule in the WT Fluc channel at F82 and F85 sites, or in the mutant channel at F82I and F85I sites (Δ*G*_1_ in Fig 3). Since there are two F82 sites and two F85 sites in Fluc, we calculated the absolute Δ*G*_bind_ of water to each of the two binding sites.

As described by Deng and Roux [74], the sampling is more efficient during calculations of absolute binding free energies if the molecule is restraint at the binding site. Therefore, to keep the water molecules at the positions indicated by the electron densities in the crystal structure, we applied position restraints to the oxygen atom of water molecules at Fluc binding sites. Likewise, we also applied position restraints on the water molecule in the pure water box. Following the scheme shown in Fig 3, the calculation of Δ*G*_1_ was separated in five sub-steps, namely, (a) $\Delta G_{1}^{\text{posre}}$, turning on the position restraints with a force constant *K*_**x**_ = 200 kJ mol^−1^nm^−2^; $\Delta G_{1}^{\text{posre}}$ was typically in the order of only 1 kJ mol^−1^, (b) $\Delta G_{1}^{\text{Coulomb}}$, turning off electrostatic, and (c) $\Delta G_{1}^{\text{LJ}}$, turning off Lennard-Jones interactions, (d) translating the non-interacting solute from the protein into a pure-solvent box, and (e) release of the position restraints in pure solvent. To obtain $\Delta G_{1}^{\text{posre}}$, 11 λ-steps were simulated for 2 ns each for all systems.

$\Delta G_{1}^{\text{Coulomb}}$ was computed from 11 λ-steps simulated for 2 ns each, and $\Delta G_{1}^{\text{LJ}}$ was computed in 41 λ-steps simulated for 2 ns each. To account for atomic overlaps occurring close to λ = 0 and λ = 1, soft-core potentials were used for Lennard-Jones interactions with *α* = 0.5, *σ* = 0.3 and a soft-core power of 1. As before, the first 50 ps of each trajectory were removed for equilibration, and the free energy differences were calculated according to Eq 1. The calculation of Δ*G*_2_ in Fig 3 was separated into the electrostatic part and Lennard-Jones interactions part, and the free energy $\Delta G_{r,x}^{\circ }$ due to applying position restraints on a non-interacting water particle was calculated analytically following ref. [75]. Accordingly, applying a translational restraining potential *U*_*r*,**x**_ with a force constant *K*_**x**_ and the form *U*_*r*,**x**_ = *K*_**x**_(**x** − **x**_0_)^2^/2 confines the molecule to a small effective volume *V*_eff_ = ∫_*V*_ exp[−*K*_**x**_(**x** − **x**_0_)^2^/2*k*_B_*T*]d**x** that can be analytically evaluated as $V_{\text{eff}}={\left(2\pi k_{\text{B}}T/K_{x}\right)}^{\frac{3}{2}}$. Hence, the corresponding free energy can be calculated via $\Delta G_{r,x}^{\circ }=-k_{\text{B}}T\ln(V^{\circ }/V_{\text{eff}})$ where *V*^∘^ = 0.02992 nm^3^ is the molecular volume of water [76]. We used *K*_**x**_ = 200 kJ mol^−1^nm^−2^, yielding $\Delta G_{r,x}^{\circ }=$ 0.774225 kJ mol^−1^.

### Maximum-likelihood estimates for the lifetime of fluoride and water molecules inside Fluc

To calculate the lifetime *τ* of the fluoride or water molecules at F82 and F85 positions inside Fluc, we used the maximum-likelihood estimate as described recently [77]. In summary, given *M* simulations of simulation time *T*_*s*_, during which *m* fluoride or water leave the binding site at times *t*_*i*_ (*i* = 1, …, *m*; *m* ≤ *M*), then the maximum-likelihood estimate *τ*_ml_ for the lifetime of fluoride or water at the binding site is given via $\tau_{\text{ml}}=m^{-1}\left[\left(M-m\right)T_{s}+\sum_{i=1}^{m}t_{i}\right]$.

### Quantum chemical calculations

The relaxed potential energy surface (PES) calculations of fluoride and benzene were carried out using density functional theory with the B3LYP functional [78], using either the ma-def2-SVP or the ma-def2-TZVP basis set [79, 80]. One additional scan was conducted using Møller-Plesset perturbation theory of second order (MP2) using the aug-cc-pVDZ basis set [81]. PES scans were computed with the ORCA 3 program system [82].

## Results

### Fluoride is less stable than water inside Fluc in free MD simulations

**Fig 4:**
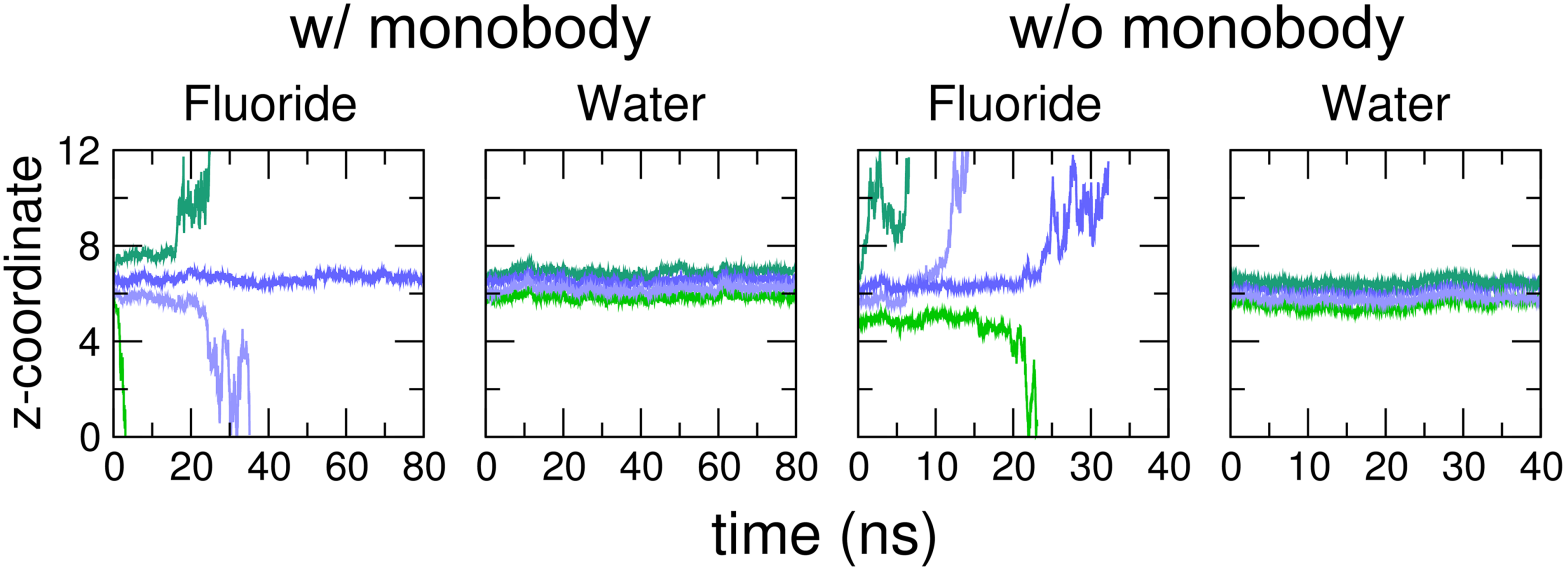
Fluoride and water molecules unbinding from the F82 and F85 sites of Fluc. Position change in *z*-direction normal to membrane in MD simulations. (A) Fluoride position inside Fluc with blocking L2 monobodies. (B) Water molecule position in Fluc with blocking L2 monobodies. (C) Fluoride position inside Fluc without monobodies. (D) Water molecule position in Fluc without monobodies. Green curves represent the molecules starting at F85 site, and blue curves belong to molecules starting at F82 site. Different shades of green and blue indicate starting positions in the two homodimers of Fluc.

The authors of the 5NKQ structure provisionally assigned fluoride ions *F*^−^ to four disconnected electron densities found in Fluc at F82 and F85 sites (Fig 1B) [36]. To test the stability of *F*^−^ inside Fluc in crystallographic and physiological conditions (*i.e.* in the presence and absence of monobodies), we carried out MD simulations with the systems depicted in Fig 1A and 1C. To obtain sufficient statistics, we carried out 30 replicas with fluoride starting at F82 and F85, among which in 10 replicas (80 ns each) Fluc was blocked by L2 monobodies (Fig 1A), while in 20 replicas (40 ns each) Fluc was free of monobodies (Fig 1C). For comparison, we performed similar simulations with water molecules starting at F82 and F85 sites. Typical time traces of the positions of water and fluoride along the channel z-axis are shown in Fig 4. Evidently, fluoride escaped earlier and more often than water in the simulations. The time traces of all the simulations are shown in S1, S2, S3 and S4 Figs. To exclude that the choice for the forcefield may influence the qualitative results, we simulated another 10 replicas of the Fluc system without monobodies using the CHARMM36 forcefield, and another 10 replicas with the polarizable DRUDE forcefield. The time traces of these simulations are shown in S5 and S6 Figs. Overall, the time traces reveal that water is more stable in the channel than fluoride.

More quantitatively, the maximum likelihood estimates for the lifetime *τ*_*ml*_ of *F*^−^ and water at the at F82 and F85 sites, as computed from all replicas, are summarized in Table 1. Overall, the lifetime of water is at least one order of magnitude longer than the lifetime of *F*^−^. This finding is most pronounced for the crystallographic conformation with monobodies, for which not a single escape event for water occurred within a total of 1,600 ns, whereas *F*^−^ escaped from the F82 and F85 sites with lifetimes of 181 ns and 25 ns, respectively (Table 1, left columns). In the absence of the monobody, Fluc was more flexible, thereby facilitating the escape of *F*^−^ and water, as evident from the reduced lifetimes (Table 1, right columns). Here, *F*^−^ escaped with lifetimes of 108 ns or 3 ns, respectively, whereas water escaped with longer lifetimes of 1,600 ns and 1,595 ns, respectively. Notably, using the CHARMM36 or the CHARMM Polarizable DRUDE force field instead of the Amber force field had only a moderate effect on lifetime of *F*^−^ in absence of the monobodies (Table 1, numbers in parentheses), rendering a major uncertainty in these findings due to the fluoride-protein interactions unlikely. In summary, during free simulations, fluoride ions were unstable at the F82 and F85 sites whereas water molecules were stable, irrespective of the presence or absence of the monobodies and irrespective of the applied force field. These findings suggest that the isolated electron densities at the F82 and F85 sites represent water and not fluoride.

**Table 1:** Maximum-likelihood estimates *τ*_*ml*_ (nanoseconds) for the lifetime of fluoride and water molecules at Fluc F82 and F85 positions.

	w/ monobody	w/ monobody	w/o monobody	w/o monobody
	fluoride	water	fluoride	water
**F82**	181	≳ 1,600	108 (91; 44)	≳ 1,600
**F85**	25	≳ 1,600	3 (3; 1)	1,595

The numbers not placed in parentheses have been calculated using the Amber ff99SB*-ILDN forcefield for the protein, and the two numbers in parentheses have been calculated using the CHARMM36 forcefield and the CHARMM polarizable DRUDE forcefield, respectively.

### Difference in binding affinity ΔΔ*G*_bind_ between fluoride and water to Fluc favors water over fluoride

Based on the free simulations, water molecules are more stable inside Fluc as compared with fluoride ions. To rationalize these findings in terms of free energies, we used thermodynamic integration (TI) to compute the difference in binding free energy ΔΔ*G*_bind_ of water versus *F*^−^ to the F82 and F85 sites of Fluc.

**Fig 5:**
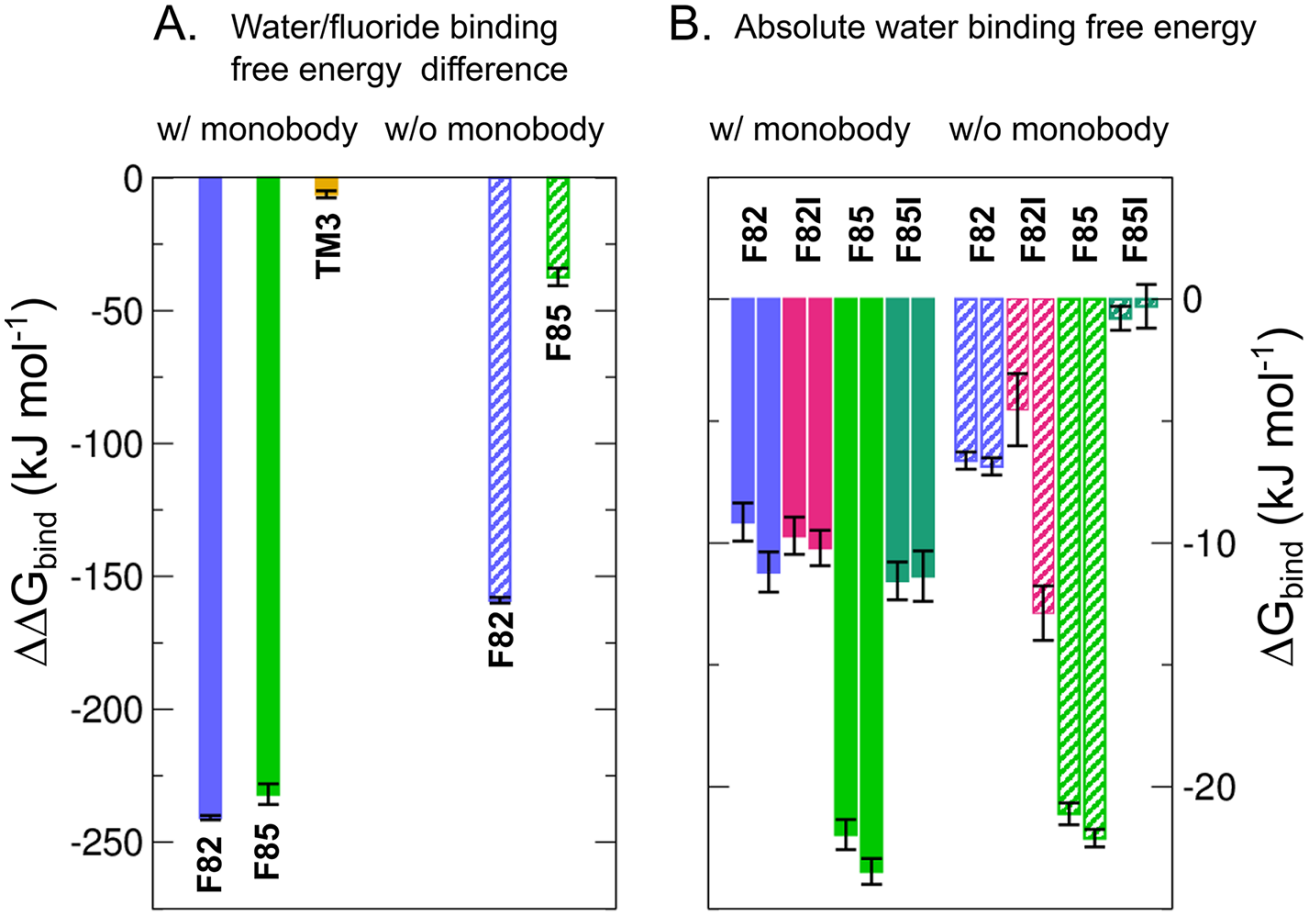
Summary of free-energy calculations. (A) Difference ΔΔ*G*_bind_ of the binding free energy of water versus an ion, defined such that a negative ΔΔ*G*_bind_ implies a more favorable binding free energy for water. Color code as follows. Blue: fluoride/water bound to F82 site; green: fluoride/water bound to F85 site; orange: sodium/water bound to TM3 site. Solid and dashed bars show results computed in the presence and absence of the L2 blocking monobodies. (B) Absolute binding free energies Δ*G*_bind_ of water to F82 (green) and F85 (blue) sites in the WT channel, and to the F82I (magenta) and F85I (dark turquoise) sites in the mutant channel. The same filled/dashed bar scheme is followed. The two values for each site correspond to the two Fluc monomers.

**Fig 6:**
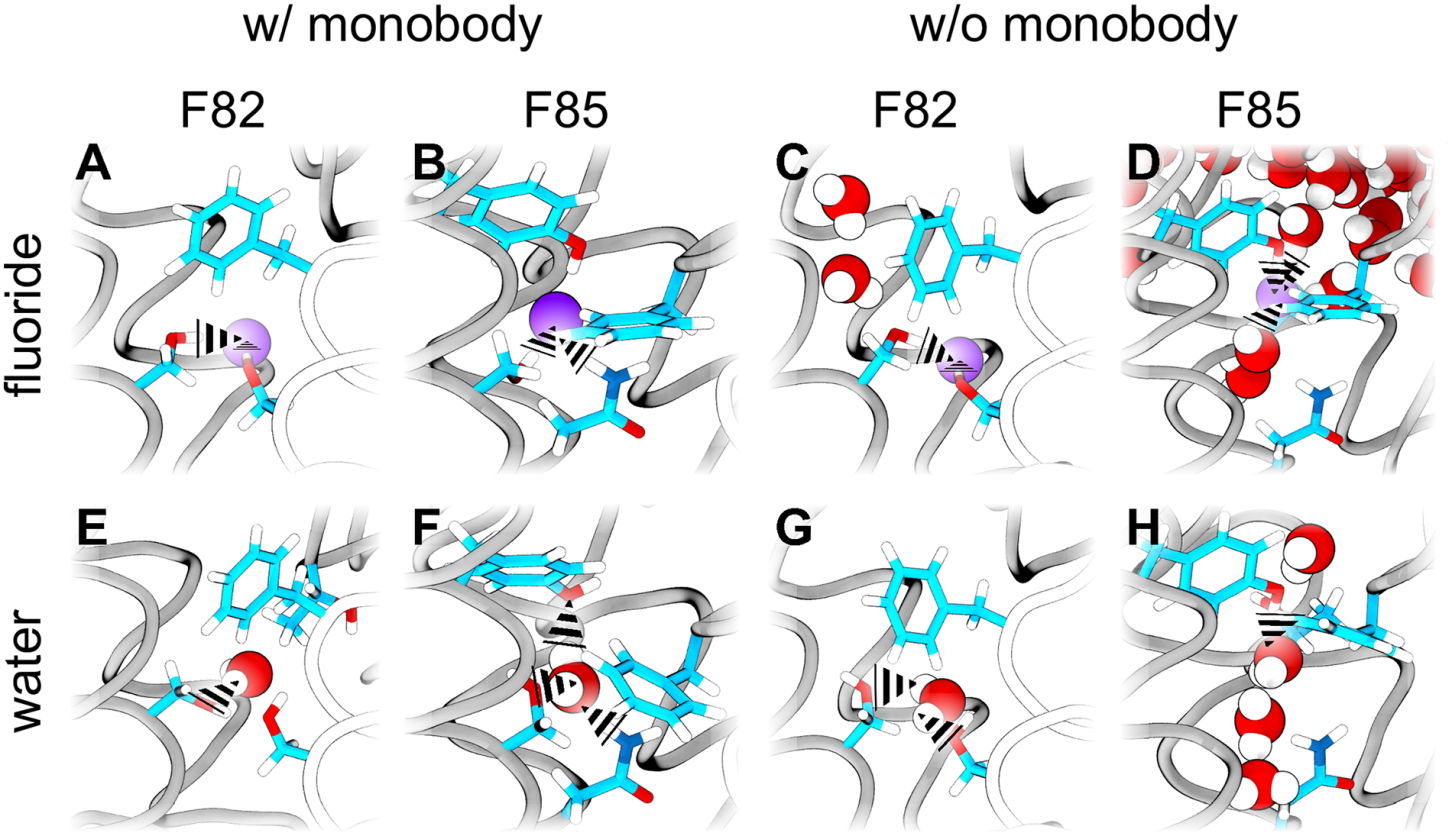
Water and fluoride at F82 and F85 sites, taken from snapshots of the relative binding free energy simulations. Several representative snapshots taken from the alchemical transformation simulations are depicted. Fluoride (A,B,C,D) is shown as purple spheres and water (E,F,G,H) as red (oxygen) and white (hydrogen) spheres. Some of the amino-acid side-chains in the F82 (A,C,E,G) and F85 (B,D,F,H) sites are depicted in colored sticks. Some of the hydrogen bonds established between the protein and the water/fluoride are highlighted with black dashed triangles.

Fig 5A shows ΔΔ*G*_bind_ as blue and green bars, where a negative value indicates a more favorable binding of water. If the L2 monobodies are blocking Fluc and thereby stabilizing the crystallographic conformation, the free energy differences at F82 and F85 sites are ΔΔ*G*_bind_ ≈ −235 kJ mol^−1^, indicating a much larger probability for water being bound at those sites as compared to *F*^−^ (Fig 5A, solid blue/green bars). These numbers may be rationalized by the large dehydration free energy for *F*^−^ of ∼500 kJ mol^−1^, which are only partly compensated by *F*^−^-protein interactions [83]. In the simulations carried out in the presence of pore-blocking monobodies, water cannot enter the pore, leaving the molecules at F82 and F85 sites dehydrated (Fig 6A, 6B, 6E and 6F). In contrast, in the absence of monobodies, we observed that *F*^−^ ions restrained at the F82 and F85 sites rapidly dragged water into the channel, thereby partly hydrating the *F*^−^ ions; subsequently, in simulations with unrestrained *F*^−^, the *F*^−^ rapidly left the channel (see Figs 4C, 6C and 6D, and Table 1). Consequently, computed ΔΔ*G*_bind_ values were less negative, in particular for the more solvent-exposed F85 site (Fig 5A, dashed blue/green bars). Nevertheless, ΔΔ*G*_bind_ still reveals a higher probability for water molecules occupying the F82 and F85 sites over fluoride anions (Fig 6G and 6H). Hence, the ΔΔ*G*_bind_ are compatible with the results from free simulations, favoring water over fluoride at the binding sites, irrespective whether antibodies are present or absent.

### Absolute binding free energy Δ*G*_bind_ of water to Fluc

The results of ΔΔ*G*_bind_ between fluoride and water indicate a much higher probability for water for being located at F82 and F85 sites as compared to fluoride. However, the ΔΔ*G*_bind_ values mainly reflect the large dehydration cost for fluoride, but they do not reveal whether water *per se* exhibits a favorable binding affinity to the F82 and F85 sites, as would be required to explain the electron densities at the F82 and F85 in terms of water. Hence, we further calculated the absolute binding free energy Δ*G*_bind_ of water to F82 and F85 sites, i.e. the free energy for translocating a water molecule from bulk solvent into the F82 and F85 sites, again either in presence or in absence of the monobodies.

Fig 5B (blue and light green bars) shows Δ*G*_bind_ of water molecules at the F82 and F85 sites in the WT Fluc channel. The results in Fig 5B are also summarized in Table 2. Evidently, both in systems with monobodies (Fig 5B, solid bars) and without monobodies (Fig 5B, shaded bars), Δ*G*_bind_ is negative, indicating a high probability for water binding to these sites. Visual inspection shows that, at the F85 sites, water may interact with the hydroxyl groups of Ser180 and Tyr104 (as hydrogen bond donor or as acceptor), and with Asn43 (as hydrogen bond acceptor), thus rationalizing the large negative Δ*G*_bind_ of approx. −20 kJ mol^−1^ (Fig 5B, light green bars). The F82 sites offer less interaction sites; here, water mainly interacts with the hydroxyl group of Ser112 and with the backbone carbonyl of Asn43, rationalizing the less negative Δ*G*_bind_ (Fig 5B, blue bars). Taken together, the coordination of the isolated densities at the F82 and F85 sites are compatible with water binding.

**Table 2:** Absolute binding free energy Δ*G*_bind_ (kJ mol^−1^) of water at F82, F85, F82I, and F85I sites.

	F82	F85	F82I	F85I
**w/ L2 monobodies**	-11.2 ± 0.8	-23.5 ± 0.5	-10.2± 0.7	-11.4 ± 1
	-9.1 ± 0.8	-22 ± 0.6	-9.7 ± 0.8	-11.6 ± 0.8
**w/o L2 monobodies**	-6.6 ± 0.4	-22.1 ± 0.4	-12.9 ± 1.1	-0.3 ± 0.9
	-6.9 ± 0.4	-21.1 ± 0.4	-4.5 ± 1.5	-0.8 ± 0.5

The results in Fig 5B and Table 2 reveal that Δ*G*_bind_ of water to F85 site is lower than Δ*G*_bind_ to F82 site regardless the absence or presence of L2 monobodies.

### Rationalizing the disappearence of electron densities in the F82I/F85I mutant

Last *et al.* showed that, in the non-conducting F80I/F83I Fluc-*Ecl* mutant, the disconnected electron densities associated to F80I and F83I sites (analogues of F82 and F85 sites in Fluc-*Bpe*) disappear [40]. The correlation between the (i) disappearance of the electron densities and (ii) loss of function was interpreted as an indication that the electron densities represent functionally relevant fluoride binding sites. Hence, to test whether the assignment of water molecules to the binding sites, as suggested by the free energy calculations reported above, is compatible with the disappearance of the electron densities in the mutant, we further computed the absolute Δ*G*_bind_ of water to F82I and F85I sites in Fluc-*Bpe*.

**Fig 7:**
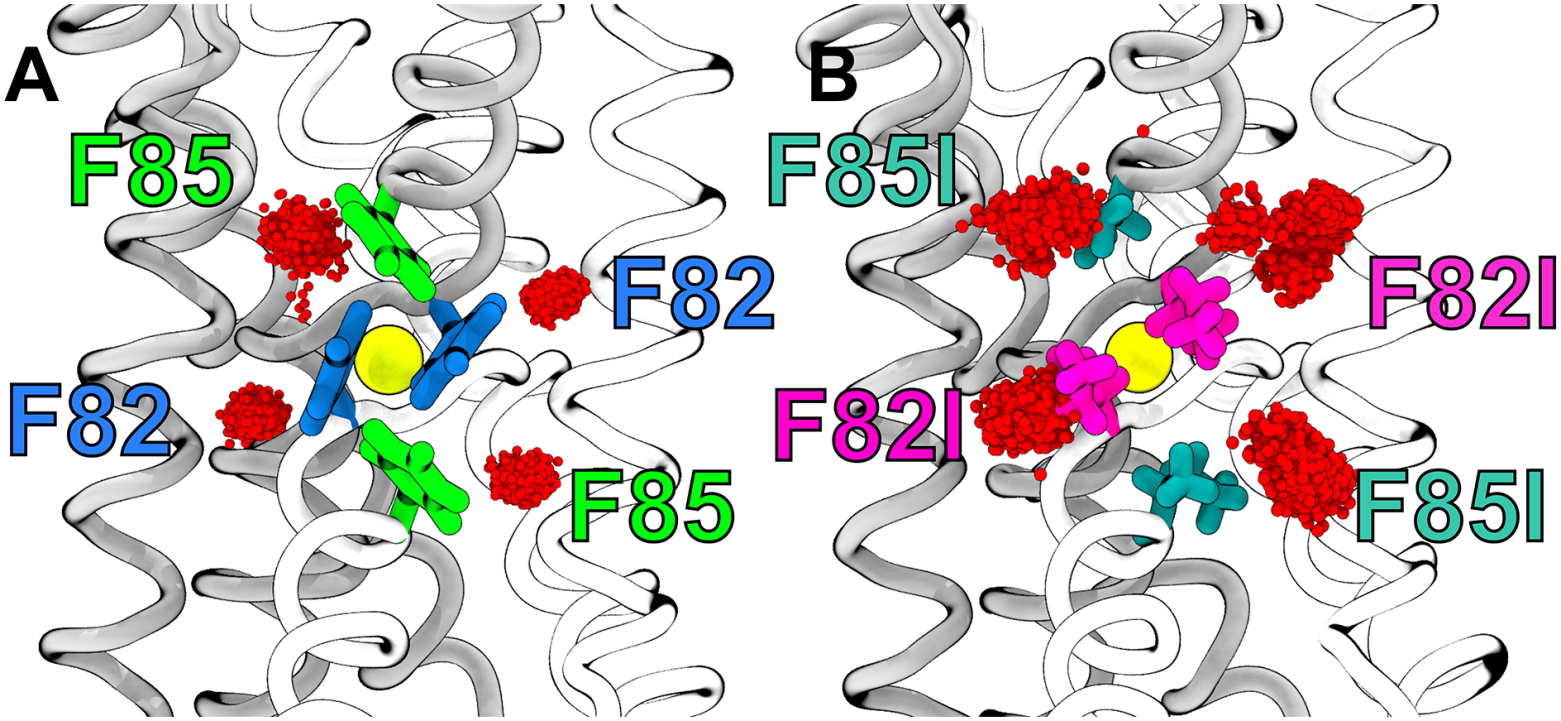
On the disappearance of electron densities at the F85 and F82 in the mutant crystals. Water oxygen atoms (red spheres) positions in the 2000 snapshots taken from 100-nanosecond simulations. (A) WT Fluc channel is shown in grey and white ribbons. The F82 and F85 binding sites are highlighted in both monomers. (B) Mutant Fluc channel is depicted as before. Here, the F82I and F85I mutated residues are highlighted.

Fig 5B (magenta and dark green bars) shows that most of the Δ*G*_bind_ values remain negative upon mutation, suggesting that the sites may be occupied also in the mutants. An exception is the F85I site without monobody; here, as soon as the analyzed water molecule is moved to bulk, it may rapidly be replaced by bulk water, leading to Δ*G*_bind_ near zero. These findings might seem to contrast the loss of electron density in the mutant crystals. Hence, we speculated that the disappearance of the electron densities in the mutants might not reflect a loss of water binding, but instead reflect increased disorder of bound water molecules in the crystal, as the Phe→Ile mutations increase the volume of the cavities. To test this hypothesis, we ran free MD simulations for 80 ns of water at the F82 and F85 sites, and we compared disorder of the water molecules between WT and mutant. Fig 7A and 7B show positions of the water oxygen atoms taken from ∼2000 MD snapshots in WT and mutant channels. To quantify the volume sampled by water molecules at each site, we computed the root mean squared fluctuations (RMSF) of water oxygen atoms at the F82 and F85 sites in each simulation, taking the mean oxygen position as reference in each case. The average RMSF of oxygen atoms at F82 and F85 sites in WT Fluc is 0.032 nm, and 0.038 nm, respectively, characterizing a highly localized water molecule. In the mutant channel, the average RMSF of oxygen atoms at F82I and F85I is increased to 0.066 nm, and 0.063 nm, respectively. Hence, water is spatially more dispersed in the mutant as compared to WT, which may rationalize that such bound water cannot be resolved in the crystal. As such, also the disappearance of the electron densities in the F82I and F85I seems compatible with water binding to these sites.

### Sodium binding to the TM3 site stabilizes its structure

The isolated density at the TM3 site of Fluc is coordinated by four main chain carbonyl oxygen atoms, and it was hence modeled as a sodium ion Na^+^ in the 5NKQ structure [36]. As a control, we tested whether MD simulations support the assignment of Na^+^ to this density. Hence, we computed the relative binding free energy difference ΔΔ*G*_bind_ between Na^+^ and water at the at TM3 site (Fig 1B, yellow sphere). Fig 5A shows a ΔΔ*G*_bind_ = −6.2 ± 1.4 kJ mol^−1^, which would indicate a weak preference of water over Na^+^. However, because (i) ΔΔ*G*_bind_ is close to zero, and (ii) protein-ion interactions still exhibit increased uncertainties in modern force fields, these findings are hardly significant. As such, free energy calculations alone are in this case insufficient to assign the electron densities.

**Fig 8:**
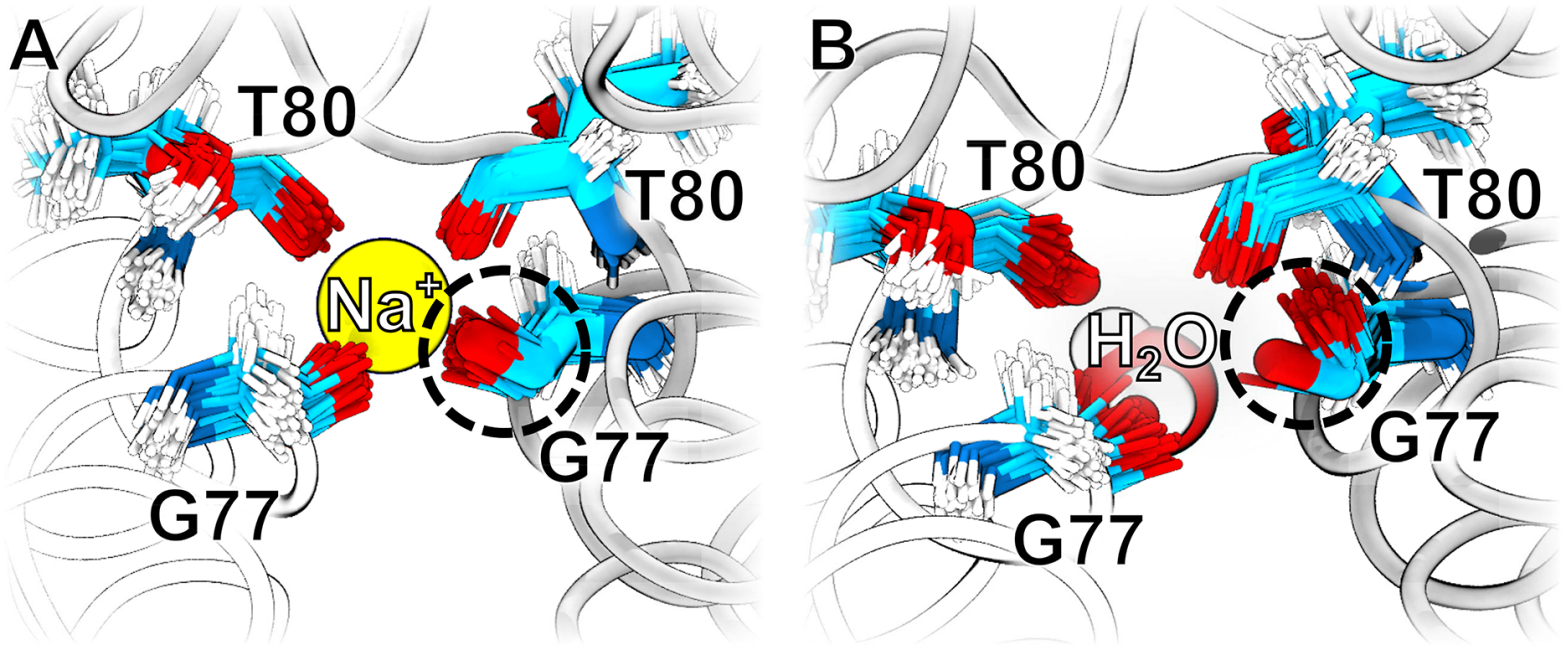
G77 and T80 residue positions in MD simulations. (A) Positions of G77 and T80 residues in a 10 ns MD simulation in which a sodium ion is at the TM3 site. The positions of G77 and T80 residues are shown as thin sticks in which oxygen is colored in red, hydrogen in white, nitrogen in blue, and carbon in cyan. The positions of the G77 and T80 residues in the crystal structure are depicted in thick sticks following the same color scheme. The protein secondary structure is showed in white and gray ribbons. (B) Positions of G77 and T80 residues in a 10 ns MD simulation in which a water molecule is at the TM3 site. The G77 residue that adopts a significantly different orientation in the simulation compared with the crystal structure is highlighted in a dashed circle.

Consequently, we instead investigated the stability of the coordination shell in free simulations with either water or Na^+^ at the TM3 site. Fig 8A and 8B show an overlay of the snapshots of G77 and T80 residues forming the TM3 site taken from free 10-nanosecond MD simulations (thin sticks), compared with the positions of G77 and T80 in the crystal structure (thick sticks). Evidently, throughout the 10 ns simulation with Na^+^, the G77 and both T80 residues remain close to the crystal structure positions (Fig 8A). In contrast, during the simulation with water at the TM3 site, the G77 and T80 reveal an increased flexibility, and G77 of one chain even deviates systematically from the crystallographic position (Fig 8B, circle). Visual inspection of the trajectories shows that the carbonyl group of G77 formed a hydrogen bond with the neighboring T80 residue because water can not form favorable contacts simultaneously with all four carbonyl groups, as pointed by the authors of the 5NKQ structure [36]. In conclusion, whereas free energy calculations are on the TM3 site an inconclusive, free simulations suggest that Na^+^ as the more likely assignment as compared to water.

### The role of the force field in modeling fluoride-phenylalanine interactions

**Fig 9:**
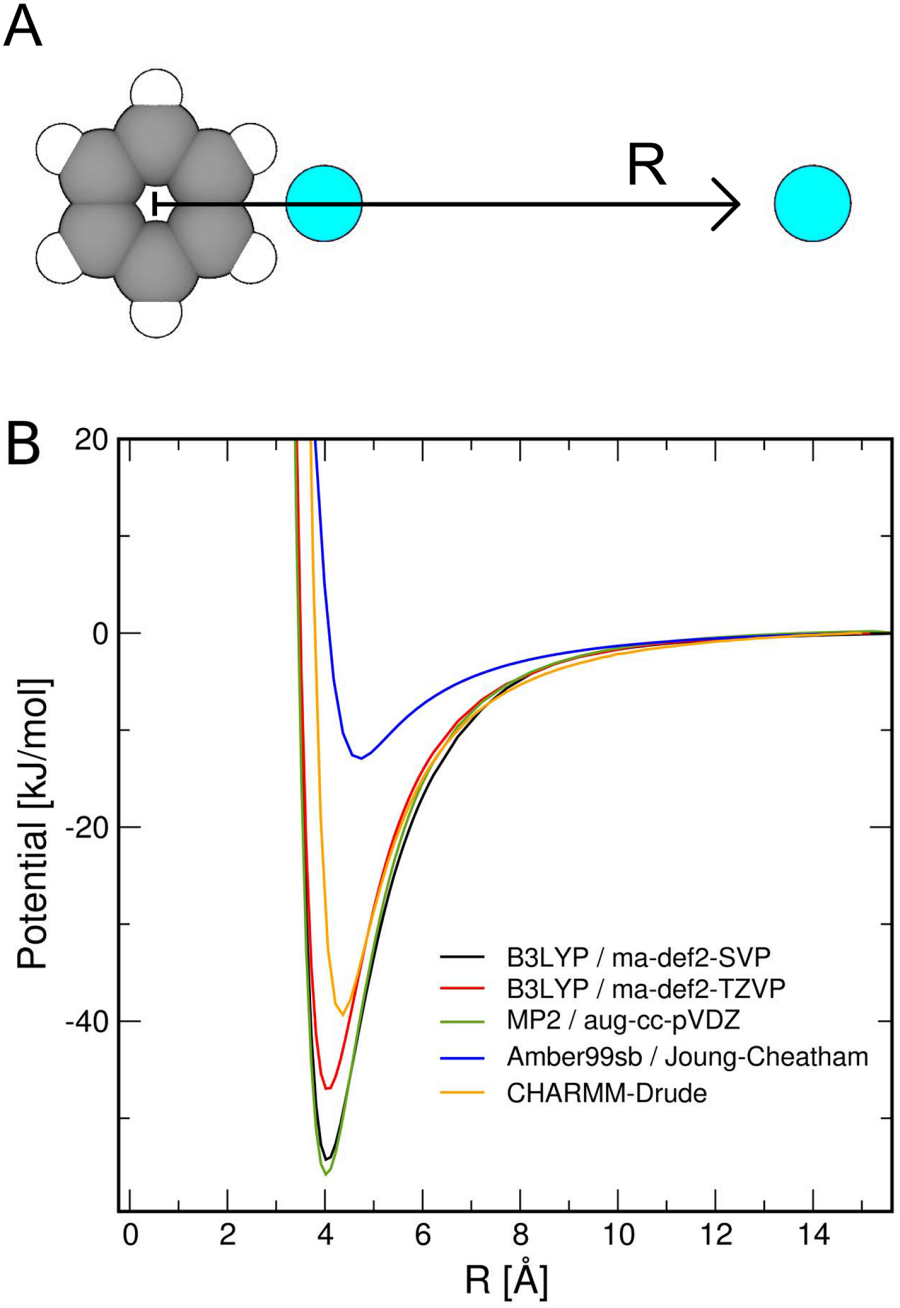
Relaxed potential energy scans (PESs) of fluoride and benzene. (A) Illustration of the coordinate *R* as the distance of fluoride (cyan sphere) from the center of mass of the benzene ring (green/white spheres), taken in the plane of the ring. The left cyan sphere indicates the position of the potential energy minimum. (B) Relaxed PESs using quantum-chemical calculations at different quantum levels and using different basis sets, as indicated in the legend (black, red, and green curves), revealing a potential energy minimum of ∼ −50 kJ mol^−1^. The non-polarizable (additive) Amber99sb force field with ion parameters by Joung and Cheatham [43] strongly underestimate fluoride-benzene interactions (blue). In contrast, the polarizable CHARMM-Drude force field [57–59] provides a PES in reasonable agreement with quantum-chemical calculations.

To test whether the main conclusions might be affected by force field uncertainties, we have computed relaxed PESs between fluoride and benzene using quantum-chemical calculations, and we compared the scans with results from an additive and from a polarizable force field. For the force field-based PESs, benzene was modeled from atoms of the phenyl ring of phenylalanine. As shown in Fig 9, quantum-chemical calculations reveal strong interactions of fluoride with the quadrupole of benzene, leading a potential energy of binding in vacuum of ∼ −50 kJ mol^−1^. The non-polarizable (additive) Amber99sb force field together with ion parameters by Joung and Cheatham strongly underestimate fluoride-phenyl interactions (Fig 9, blue curve). This suggests that the relative binding free energies between fluoride and water presented in Fig 5A overestimate the preference for water by approx. 40 kJ mol^−1^. However, because the additive force field yielded ΔΔ*G*_bind_ values in the crystallographic conformation with monobodies in the order ∼−235 kJ mol^−1^, such correction will not change the qualitative findings of the free energy calculations. Compared to the non-polarizable force field, the polarizable CHARMM-Drude force field models stronger fluoride-benzene interactions, in better agreement with the quantum-chemical calculations (Fig 9, orange curve). However, despite stronger fluoride-benzene interactions, the life time of fluoride at the F82 and F85 sites during CHARMM-Drude simulations was even shorter as compared to Amber-based simulations (Table 1, second numbers in parentheses), suggesting that stability assessments in this case do not critically depend on the fluoride-phenyl interactions.

## Discussion

Ions, water, and other small molecules play key roles in the structure and function of many proteins, taking part in activation and de-activation processes, in enzymatic reactions, and in the formation and stabilization of structural motifs. Hence, the correct assignment of disconnected electron densities in crystal structures forms the basis for deriving functional mechanisms from structural data. However, the assignment of densities is in many cases far from obvious [9–17, 84, 85].

The present study suggests that free-energy calculations and free MD simulations may aid the assignment of disconnected electron densities in the crystal structure of proteins. More specifically, three simulation methods may serve for this purpose:

1. Relative binding free energies ΔΔ*G*_bind_ between two putative molecular species, such as between an ion and water, reveal which species binds with higher affinity to the site of the disconnected electron density. Hence, ΔΔ*G*_bind_ values in principle clarify which interpretation of the density is more plausible. However, if the computed ΔΔ*G*_bind_ involves the binding of ions, ΔΔ*G*_bind_ is dominated by strong ion-water and, possibly, strong ion-protein interactions, leading to increased uncertainties due to imperfections in current force fields (see below). Hence, purely ΔΔ*G*_bind_ values that strongly deviate from zero, as found for the fluoride/water alchemical transformations found here, allow for robust conclusions.
2. Given that ΔΔ*G*_bind_ favor the binding of a species A over another species B, absolute binding affinities Δ*G*_bind_ (or bulk to protein transfer free energies) reveal whether the binding of the species A is plausible *per se*. The binding of species A with high probability, such that A is visible in the crystal, requires Δ*G*_bind_ to be negative or near zero. As a side remark, the fraction of binding sites occupied by species A depends, apart from Δ*G*_bind_, also on the bulk concentration of A.
3. The stability of structures in short free MD simulations may provide additional indications on the nature of electron densities. In particular, rapid and reproducible drifts from the crystal structure hint towards incorrect assignments of densities, or towards other problems in the structure. For instance, here we found pronounced instability of F^−^ ions at the F82 and F85, irrespective of the applied force field (Fig 4, S1–S4 Figs), and we observed a drift in the cage of the TM3 site upon assignment of a water molecule to this site (Fig 8). In turn, however, the stability of a structure in short simulations does not necessarily imply that the assignment is correct, since structural drifts may occur in a stochastic manner and on longer time scales as compared to the simulation time. Therefore, conclusions can only be drawn from comparative stability assessments and from multiple independent simulations.

Here, we illustrated these methods by analyzing the five disconnected electron densities present in the crystal structure of the fluoride channel Fluc. In summary, the free-energy calculations and stability assessments suggest that four of the disconnected densities represent water molecules but not fluoride ions, in contrast to a provisional assignment by the authors of the crystal structure. For the fifth, central density, the results from free-energy calculations were inconclusive; however, stability assessments confirm the assignment of a sodium ion, as previously suggested [36].

The accuracy of the absolute and relative binding affinities depends on the accuracy of the applied force fields. Most critically, ion-protein interactions in additive force fields are still a source of uncertainty. For instance, the potential energy surface (PES) along a sodium-carboxylate distance in vacuum computed by older force fields may deviate from quantum-chemical calculations by tens of kilojoule per mole, which has triggered extensive force field developments in recent years [70–72]. For the present study on Fluc, we compared the interactions between fluoride and the quadrupolar field of a benzene ring computed by quantum-chemical calculations with the predictions by a non-polarizable (Amber99sb) and a polarizable (CHARMM-Drude) force field (Fig 9). The PES derived with CHARMM-Drude agreed reasonably with the PESs from quantum-chemical calculations; in contrast, Amber99sb combined with the Joung/Cheatham ion model [43] strongly underestimates fluoride–phenylalanine interactions, leaving room for future force field improvements. However, the qualitative findings of this study are not affected by these force field imperfections because (i) simulations with CHARMM-Drude confirmed the instability of F^−^ at the F82 and F85 sites, and (ii) relative binding free energy differences between F^−^ and water are by far larger than the uncertainties imposed by the non-polarizable force fields.

In this work, simulations have been carried out in a physiologically realistic environment including a lipid bilayer, explicit solvent molecules, and counter ions. During the alchemical transformations, this setup allowed us to maintain the overall charge neutrality of the system by simultaneously morphing water to fluoride in bulk while morphing fluoride to water in the protein. Maintaining charge neutrality avoids that a so-called background charge density is built up during the alchemical transformation, which is a consequence of the particle-mesh Ewald (PME) method for computing electrostatic interactions [86].

To carry out the ΔΔ*G*_bind_ calculations used here in a high-throughput fashion, it would be desirable to avoid the setup of a physiologically realistic simulation system, but to simulate the crystallographic unit cell instead. Because the unit cell might not contain sufficient volume of bulk water for placing a counter charge, such setup may require alchemical transformations that do not maintain charge neutrality, leading to artifacts due the interactions of ions with the background charge. For solvation free energy calculations of ions in a uniform solvent, analytic corrections for background charge effects are available (in addition to effects due to finite size of the simulation system and finite volume of the ion) [87–95] In addition, corrections for inhomogeneous systems of simple geometry, such as hydrophobic slaps or spheres in water, have also been proposed [86]. Corrections for more complex inhomogeneous systems, such as protein crystals, are to our knowledge not yet established, but approximations might build upon previous related work.

## Supporting information

S1 Fig Fluoride ion positions in Fluc blocked by L2 monobodies. Green and blue curves represent the molecules starting at the F85 and F82 sites, respectively. Different shades of green and blue indicate starting positions in the two homodimers of Fluc.
(EPS)

S2 Fig Water molecule positions in Fluc blocked by L2 monobodies. Green and blue curves represent the molecules starting at the F85 and F82 sites, respectively. Different shades of green and blue indicate starting positions in the two homodimers of Fluc.
(EPS)

S3 Fig Fluoride ion positions in Fluc free of monobodies. Green and blue curves represent the molecules starting at the F85 and F82 sites, respectively. Different shades of green and blue indicate starting positions in the two homodimers of Fluc.
(EPS)

S4 Fig Water molecule positions in Fluc free of monobodies. Green and blue curves represent the molecules starting at the F85 and F82 sites, respectively. Different shades of green and blue indicate starting positions in the two homodimers of Fluc.
(EPS)

S5 Fig Fluoride ion positions in Fluc free of monobodies, simulated with the CHARMM36 force field for the protein and Joung/Cheatham parameters for fluoride. Green and blue curves represent the molecules starting at the F85 and F82 sites, respectively. Different shades of green and blue indicate starting positions in the two homodimers of Fluc.
(EPS)

S6 Fig Fluoride ion positions in Fluc free of monobodies, simulated with the CHARMM-Drude force field for the protein and the polarizable fluoride model by Lamoureux et al. Green and blue curves represent the molecules starting at the F85 and F82 sites, respectively. Different shades of green and blue indicate starting positions in the two homodimers of Fluc.
(EPS)
